# *Sprouty1*, a new target of the angiostatic agent 16K prolactin, negatively regulates angiogenesis

**DOI:** 10.1186/1476-4598-9-231

**Published:** 2010-09-02

**Authors:** Céline Sabatel, Anne M Cornet, Sébastien P Tabruyn, Ludovic Malvaux, Karolien Castermans, Joseph A Martial, Ingrid Struman

**Affiliations:** 1 Unit of Molecular Biology and Genetic Engineering, GIGA-research, University of Liège, B34, Avenue de l'Hôpital, 1, B-4000 Liège, Belgium

## Abstract

**Background:**

Disorganized angiogenesis is associated with several pathologies, including cancer. The identification of new genes that control tumor neovascularization can provide novel insights for future anti-cancer therapies. *Sprouty1 (SPRY1)*, an inhibitor of the MAPK pathway, might be one of these new genes. We identified *SPRY1 *by comparing the transcriptomes of untreated endothelial cells with those of endothelial cells treated by the angiostatic agent 16 K prolactin (16 K hPRL). In the present study, we aimed to explore the potential function of *SPRY1 *in angiogenesis.

**Results:**

We confirmed 16 K hPRL induced up-regulation of *SPRY1 *in primary endothelial cells. In addition, we demonstrated the positive *SPRY1 *regulation in a chimeric mouse model of human colon carcinoma in which 16 K hPRL treatment was shown to delay tumor growth. Expression profiling by qRT-PCR with species-specific primers revealed that induction of *SPRY1 *expression by 16 K hPRL occurs only in the (murine) endothelial compartment and not in the (human) tumor compartment. The regulation of *SPRY1 *expression was NF-κB dependent. Partial *SPRY1 *knockdown by RNA interference protected endothelial cells from apoptosis as well as increased endothelial cell proliferation, migration, capillary network formation, and adhesion to extracellular matrix proteins. *SPRY1 *knockdown was also shown to affect the expression of *cyclinD1 *and *p21 *both involved in cell-cycle regulation. These findings are discussed in relation to the role of SPRY1 as an inhibitor of ERK/MAPK signaling and to a possible explanation of its effect on cell proliferation.

**Conclusions:**

Taken together, these results suggest that SPRY1 is an endogenous angiogenesis inhibitor.

## Background

Many growth factors including vascular endothelial growth factor (VEGF) and basic fibroblast growth factor (bFGF), in association with their receptor tyrosine kinase (RTK) receptors, play a crucial role in angiogenesis in normal and pathological settings [1]. Essential to most RTK-mediated signaling is the activation of the extracellular-signal-regulated kinase/mitogen-activated protein kinase (ERK/MAPK) signaling cascade. This cascade is precisely controlled by the activity of various regulatory proteins [2], including members of the Sprouty (SPRY) protein family.

SPRY was originally described as an antagonist of Breathless FGF receptor signaling during tracheal branching in Drosophila [3]. Four mammalian homologs (*SPRY1-4*) have been described and are widely expressed in embryonic and adult tissues, except for *SPRY3 *whose expression is believed to be restricted to the brain and testes in adults [4]. All SPRY proteins share a highly conserved, cysteine-rich C-terminal domain and a more variable N-terminal domain. They are subject to tight control at multiple levels: differential localization, post-translational modification, and regulation of protein levels. SPRY specifically inhibits RTK-mediated Ras-Erk/MAPK signaling. At which stage SPRY blocks ERK/MAPK activation remains controversial, and evidence to date suggests the existence of multiple mechanisms that depend on the cell context and/or the identity of the RTK [5-7]. Due to their inhibitory activity on the ERK/MAPK pathway, SPRY generally acts as a tumor suppressor. Recently, the anti-tumor potential of SPRY4 was shown to be associated with its ability to inhibit angiogenesis [8]. Moreover, the angiostatic activity of both SPRY2 and SPRY4 has also been demonstrated *in vivo *in a mouse model of ischemia [9].

Our laboratory and others have identified 16 K prolactin (16 K hPRL), the 16-kDa N-terminal fragment of human prolactin, and its derived peptides as very potent angiostatic compounds both *in vitro *and *in vivo *[10,11]. 16 K hPRL is able to inhibit tumor growth and metastasis in various mouse models by inhibiting neovascularization [12-15]. The potential therapeutic use of 16 K hPRL has also been observed in non-cancer pathological models like retinopathy [16]. Postpartum cardiomyopathy, a disease characterized by acute heart failure in women in the late stage of pregnancy up to several months postpartum, has been shown to be a consequence of an excessive production of 16 K hPRL [17]. To date, the mechanisms by which 16 K hPRL inhibits angiogenesis have only been partially elucidated. In bovine endothelial cells, the angiostatic activity of 16 K hPRL appears to be mediated by a saturable high-affinity binding site distinct from the PRL receptor [18]. 16 K hPRL triggers endothelial cell apoptosis by activation of nuclear factor κB (NF-κB) [19,20]. In addition, 16 K hPRL induces endothelial cell cycle arrest in G_0_-G_1 _and G_2_-M [21], in parallel with inhibition of bFGF and VEGF stimulated MAPK activation [22]. More recently, we identified an important link between 16 K hPRL and the immune system using a transcriptomic analysis performed on 16 K hPRL-treated endothelial cells. 16 K hPRL induces leukocyte adhesion to endothelial cells by activating NF-κB [23].

Interestingly, *SPRY1 *was amongst the targets of 16 K hPRL found in the aforementioned transcriptomic study. SPRY1 has been implicated in the inhibition of bFGF and VEGF-induced proliferation and differentiation *in vitro *[24], however the physiological role of SPRY1 in angiogenesis still remains to be elucidated. Here, after confirming upregulation of *SPRY1 *expression by 16 K hPRL both *in vitro *(in primary endothelial cells) and *in vivo *(in a mouse xenograft tumor model), we performed *SPRY1*-knockdown experiments to test the possible involvement of *SPRY1 *in regulating angiogenesis. Indeed, *SPRY1 *emerges as a novel endogenous angiogenesis inhibitor with potential applicability in the clinic.

## Results

### 16 K hPRL treatment increases *SPRY1 *mRNA and protein levels in primary and human endothelial cells

A previously performed differential transcriptomic study on ABAE (Adult Bovine Aortic Endothelial) cells cultured with or without the angiostatic compound 16 K hPRL, revealed 216 genes which were differentially expressed [23]. From these 216 genes, we selected 2-fold up-regulated *SPRY1 *as a potential new angiogenesis regulator, notably because of its function in cell proliferation.

**Figure 1:**
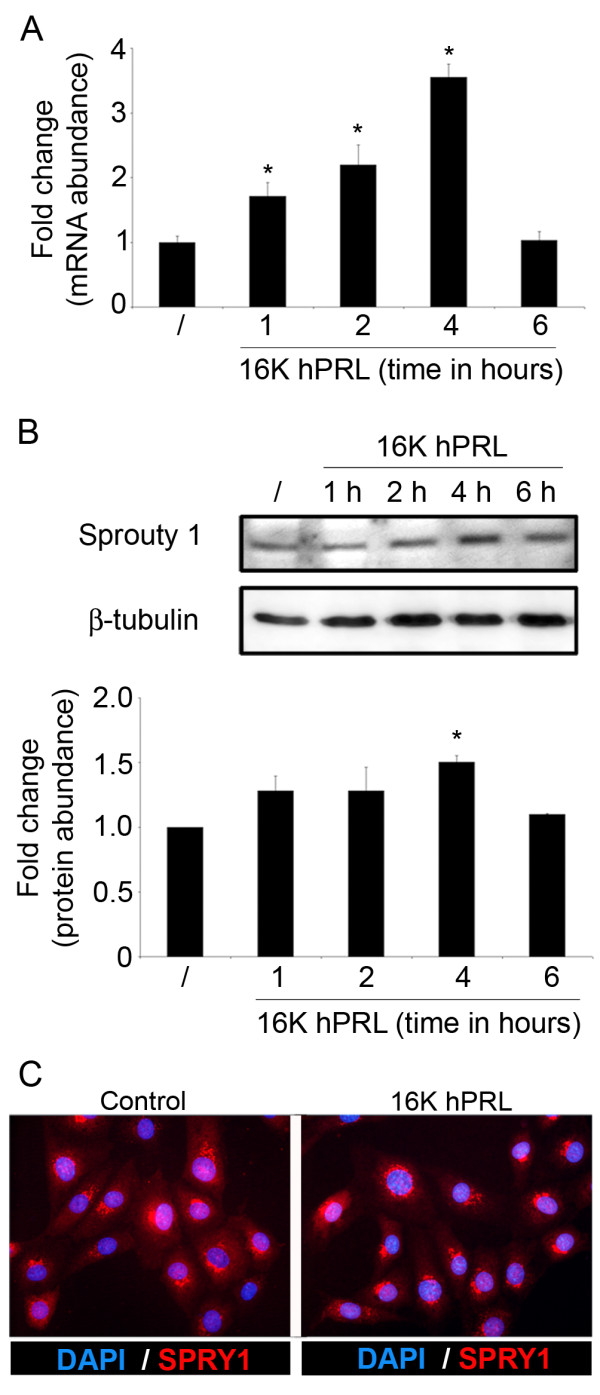
**16 K hPRL treatment increases *SPRY1 *mRNA and protein levels in primary and human endothelial cells**. **A**. *SPRY1 *mRNA expression measured by qRT-PCR in RNA extracted from ABAE cells incubated for 1 to 6 hours with 16 K hPRL (10 nM). Data were normalized to the *GAPDH *transcript level. **B**. SPRY1 protein expression analyzed by Western blotting on total protein extracted from ABAE cells treated for 1 to 6 hours with 16 K hPRL (10 nM). The level of beta-tubulin was measured as an internal control. Bottom panel: quantification of the top panel. A densitometry analysis was performed using ImageJ software and showed the SPRY1/beta tubulin intensity. **C**. The subcellular localization of SPRY1 was determined in ABAE cells by immunofluorescence microscopy using an antibody specific for SPRY1 (red) and a dapi staining for nuclei (blue) **A**, **B**. Mean fold change versus untreated cells (/) is reported with the corresponding SD (n = 3).*: significant at p < 0.05. The results shown are representative of at least three independent experiments.

We first confirmed the results of the transcriptomic analysis by performing a time response analysis of *SPRY1 *mRNA expression in ABAE. 16 K hPRL treatment (10 nM) of ABAE cells induced the expression of *SPRY1 *in ABAE over time, with a maximum up-regulation 4 h post-treatment. *SPRY1 *expression returned to base levels after 6 h of 16 K hPRL treatment (Fig 1A). This regulation was confirmed at the protein level since SPRY1 protein levels increase gradually after treatment with 16 K hPRL, reaching a maximum after 4 h (Fig 1B).

*SPRY1 *expression was also analyzed in a human endothelial cell line. In HMVECs (human microvascular endothelial cells), the *SPRY1 *mRNA level was undetected under basal conditions. However, low levels of *SPRY1 *mRNA appeared after 16 K hPRL treatment (data not shown). Unfortunately, the fold induction was thus not possible to determine in this case and the expression level of *SPRY1 *in HMVECs was too low to be detected by Western blotting.

To determine whether 16 K hPRL modulates the subcellular localization of SPRY1 in endothelial cells, we performed an immunofluorescent staining on ABAE cells. In untreated cells, SPRY1 was distributed throughout the cells; especially in the perinuclear regions. This was not changed after 16 K hPRL treatment (Fig 1C) indicating that 16 K hPRL does not seem to affect SPRY1 localization.

### 16 K hPRL increases endothelial *SPRY1 *expression *in vivo *in a mouse xenograft tumor model

**Figure 2:**
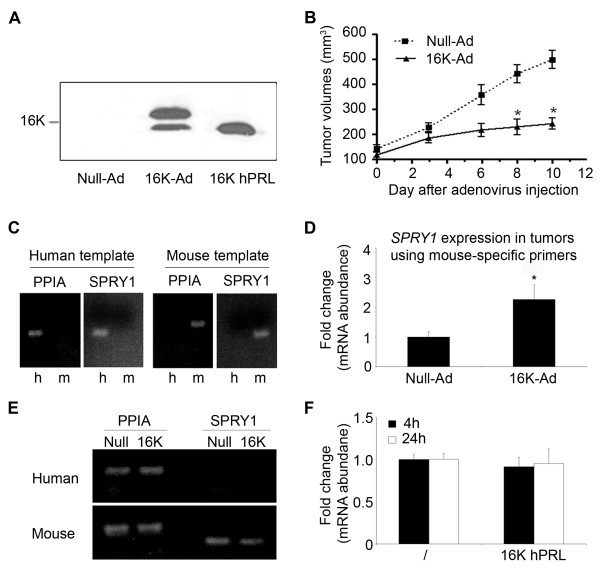
**16 K hPRL increases endothelial *SPRY1 *expression *in vivo *in a mouse xenograft tumor model**. Effect of 16 K hPRL on tumor growth. Representative data of 2 independent experiments are shown. **A**. Western blotting performed on protein extracts from tumors (100 μg protein) with an anti-hPRL polyclonal antibody, recombinant 16 K hPRL (100 ng, 16 K hPRL) was used as control. **B**. Tumor growth curves of HCT116 tumor cells in mice treated with 16 K-Ad or Null-Ad. **C**. Agarose electrophoresis of products of end-point PCR performed with human-specific (h) or mouse-specific (m) primers on cloned full-length mouse or human *SPRY1 *or *PPIA (cyclophilin A) *cDNA. **D**. Analysis of *Spry1 *mRNA expression by qRT-PCR using mouse-specific primers in RNA extracted from tumors. Data were normalized with respect to the mouse *ppia *transcript level. **E**. Agarose electrophoresis of products of end-point PCR performed with human-specific or mouse-specific primers on RNA extracted from tumors harvested from nude mice injected with HCT116 tumor cells and treated with 16 K-Ad or control Null-Ad adenovirus. **F**. The *SPRY1 *mRNA expression measured by qRT-PCR in RNA extracted from HCT116 cells after 2 or 4 h of treatment with 16 K hPRL (10 nM). The data were normalized to the *PPIA *transcript level. **D, F**. Data are mean fold change + SD (n = 3), *: significant at p < 0.05. The results shown are representative of at least three independent experiments.

We further assessed the regulation of endothelial *SPRY1 *expression by 16 K hPRL *in vivo *in a mouse xenograft tumor model consisting of nude mice injected *s.c*. with human HCT116 cells. When tumors reached an average volume of 150 mm^3^, mice were treated with 16 K-Ad or Null-Ad by intra-tumoral injections. In order to verify that 16 K hPRL was synthesized in the tumors treated with this vector, Western blot analyses were performed on protein extracts obtained from 16 K-Ad- and Null-Ad-treated tumors (Fig 2A). Indeed, the 16 K-Ad-treated tumors showed high levels of two 16 K hPRL isoforms, while the two bands were absent in the Null-Ad treated tumors. As previously reported 16 K hPRL has the ability to undergo glycosylation and thus appears in multiple isoforms [16]. We detected a significantly delay in established HCT116 tumor growth after 16 K-Ad treatment compared to Null-Ad as depicted by the tumor growth curves (Fig 2B). This is for the first time that 16 K hPRL has been shown to reduce established growth of human tumor cells *in vivo*.

As the developing human tumors recruit mouse endothelial cells to form their vasculature in this model [25], it is possible to measure separately the levels of *SPRY1 *transcripts in the stromal-vascular and the tumor compartments. Therefore, we performed quantitative real time-PCR (qRT-PCR) and used respectively mouse-specific and human-specific primers. As shown in Fig 2C, the designed primers are species-specific, since false-template PCRs combining human cDNA with mouse primers or mouse cDNA with human primers failed to produce detectable amounts of amplicons. In the stromal-vascular compartment, *Spry1 *expression was found to be higher in mice treated with 16 K-Ad than in mice treated with the control vector (Fig 2D). Similar results were obtained for the other Sprouty family member *Spry2 *(see Additional file 1). No *SPRY1 *expression could be detected in the human tumor compartment even after 40 cycles of PCR amplification (Fig 2E).

We also assessed the effect of 16 K hPRL on *SPRY1 *expression in HCT116 *in vitro*. Although we were unable to detect *SPRY1 *in the tumor samples of the *in vivo *experiment, the Ct values of *SPRY1 *in the HCT116 cells *in vitro *were very high but in detection rate. In these tumor cells in culture, 16 K hPRL treatment had no effect on the mRNA expression level of *SPRY1 *neither after 4 h or 24 h of treatment with 10 nM 16 K hPRL (Fig 2F). These results suggest that 16 K hPRL treatment specifically amplifies endothelial *SPRY1 *expression.

### *SPRY1 *expression in endothelial cells is dependent of NF-κB activity

**Figure 3:**
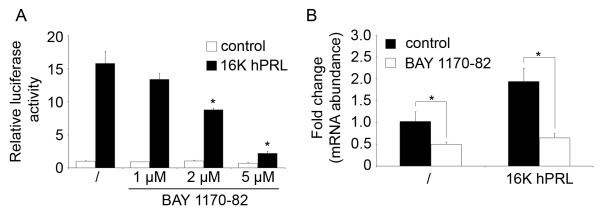
***SPRY1 *expression in endothelial cells is dependent of NF-κB activity**. **A**. Luciferase activity of ABAE cells transfected with the pElam-Luc reporter gene vector and cultured for 1 h with or without treatment with increasing concentrations of BAY 1170-82 prior to incubated for 3 h without specific treatment (control) or with 16 K hPRL (10 nM). Luciferase activities were normalized to the β-galactosidase activity. **B**. *SPRY1 *mRNA expression analyzed by qRT-PCR in RNA extracted from ABAE cells cultured with or without treatment with BAY 1170-82 (5 μM) prior to 2 h-incubation with 16 K hPRL (10 nM). Data were normalized to the *GAPDH *transcript level. Data are mean fold change + SD (n = 3), *: significant at p < 0.05. The results shown are representative of at least three independent experiments.

We have previously demonstrated a central role for NF-κB in the molecular response of 16 K hPRL in endothelial cells [23]. To assess the importance of NF-κB in 16 K hPRL-induced *SPRY1 *expression, we used the chemical inhibitor of NF-kB activation, BAY 1170-82, which interferes with IKK activation [26]. First, we transfected ABAE cells with a pElam-Luc reporter gene vector which allows specific detection of NF-κB activity. As expected, luciferase activity was increased 15 fold after 16 K hPRL treatment. This induction was reduced in a dose-dependent manner by pre-incubation of the cells with BAY 1170-82 (Fig 3A). In addition, inhibition of NF-κB activity by pre-incubating the cells with BAY 1170-82 inhibited the induction of *SPRY1 *by 16 K hPRL (Fig 3B). Interestingly, treatment of ABAE cells solely with BAY 1170-82 also significantly reduced *SPRY1 *expression in ABAE cells. These results demonstrate that the expression of *SPRY1 *in endothelial cells is dependent of NF-kB activation.

### *SPRY1 *silencing protects cells from apoptosis and induces endothelial cell adhesion, migration, and tube formation

**Figure 4:**
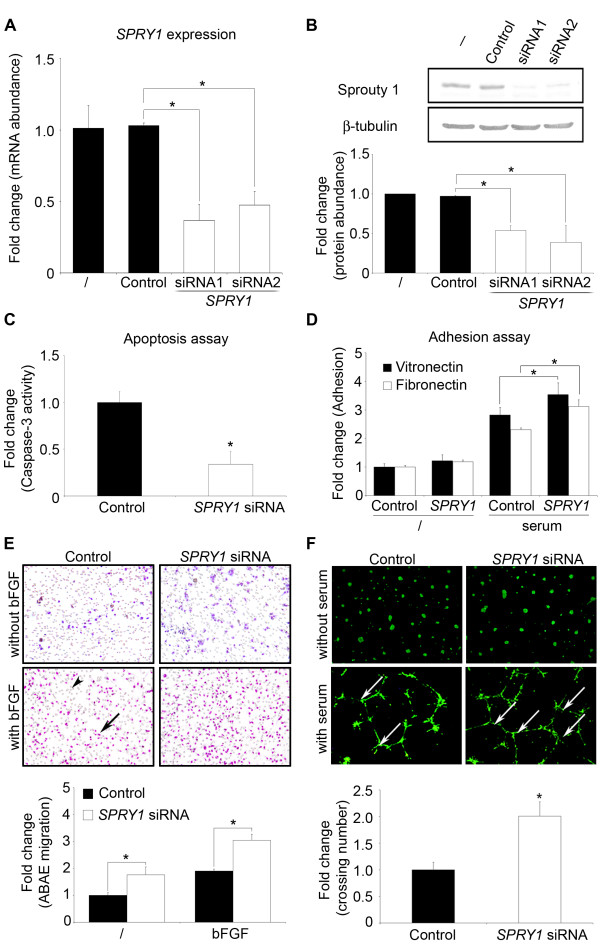
***SPRY1 *silencing protects cells from apoptosis and induces endothelial cell adhesion, migration, and tube formation**. **A**. *SPRY1 *mRNA levels quantified by qRT-PCR from ABAE cells transfected (48 h) with non-silencing siRNA (Control) or with two different *SPRY1 *siRNAs. **B**. SPRY1 protein level measured by Western blotting from ABAE cells transfected as indicated in **A**. The beta-tubulin level was also measured as an internal control. Bottom panel: quantification of the top panel: SPRY1/Beta tubulin. **C**. Apoptosis was assessed 48 hours after ABAE cell transfection with non-silencing (Control) or with a *SPRY1 *siRNA by quantification of caspase-3 activity. **D**. Effect of *SPRY1 *silencing on adhesion of ABAE cells to fibronectin or vitronectin. **E**. Migration in a modified Boyen chamber assay. 36 h after transfection cells were seeded in the upper chamber of the Boyden. Migration was assessed by counting cells on the lower face of the membrane after 16 h. Arrow head: pore of the membrane and arrow: cells stained with crystal violet. **F**. 36 h after transfection, ABAE cells were seeded on Matrigel and incubated for 16 h. Living cells were labeled with calcein-AM. Quantitative analysis of network structures was performed by measuring the number of intersections between tubes in the network (arrows). **E, F: **Top panel: representative images for migration and tube network formation assays. Bottom panel: Quantification. **A-F: **Data are mean fold + SD (n = 3). The results are representative of at least three independent cell transfections. *: significant at p < 0.05.

To investigate the specific function of *SPRY1 *in endothelial cells, we used small interfering RNA. ABAE cells transfected with 50 nM of *SPRY1 *siRNA duplexes demonstrated a significant reduction of *SPRY1 *mRNA levels 48 h post-transfection. We tested two different *SPRY1 *siRNA duplexes which both lead to a 60% decline of *SPRY1 *mRNA levels in endothelial cells compared to a control-siRNA (Fig 4A). This was confirmed at the protein level by Western blotting on cell extracts obtained 48 h post-transfection (Fig 4B). The tested siRNA constructs were specific for *SPRY1 *and did not effect the expression of the other Sprouty family members *SPRY2 *and *SPRY4 *(see Additional file 2 - *SPRY2 *and *SPRY4 *expression after *SPRY1 *silencing). Expression of *SPRY3 *was not detected in ABAE cells. Both siRNA duplexes directed against *SPRY1 *were used in the functionality assays on primary endothelial cells 48 h post-transfection.

Since *SPRY1 *expression is regulated by NF-κB activation and NF-κB is shown to be involved in endothelial cell apoptosis by activation of caspase-3 [20], we first investigated a possible role for *SPRY1 *in endothelial cells in this process. Activation of the effector protease caspase-3 is one of the most common events in the apoptotic signaling pathway. *SPRY1 *knockdown was found to reduce caspase-3 activity in endothelial cells by 60% as compared to the activity measured in cells transfected with the control siRNA duplex (Fig 4C). Similar results were obtained with both siRNA duplexes (data not shown). Thus, we can conclude that a decreased expression of *SPRY1 *protects endothelial cells from apoptosis.

Next we tested the effect of decreased *SPRY1 *expression in several other angiogenesis related processes. Interactions of endothelial cells with the extracellular matrix (ECM) are crucial, as endothelial cells are anchorage-dependent in numerous physiological processes. We examined the adhesion of transfected endothelial cells on 2 major ECM components vitronectin and fibronectin. Forty-eight hours after transfection with a *SPRY1 *siRNA duplex or with the non-silencing control siRNA duplex, the level of adhesion on vitronectin or fibronectin was slightly but significantly higher in cells where *SPRY1 *was silenced (Fig 4D). These data suggest that *SPRY1 *knockdown increases endothelial cell adhesion to ECM proteins.

Once endothelial cells have adhered, cells degrade the ECM which allows migration of the cells. We assessed the effect of *SPRY1 *silencing in endothelial cells on cell migration *via *a modified Boyden chamber with cells collected 48 h post-transfection. bFGF was used as chemoattractant for the endothelial cells. In this experiment cells transfected with the *SPRY1 *siRNA duplex showed a 70% greater migration capacity than control-duplex-transfected cells in the absence of bFGF. When bFGF was added to stimulate cell migration, an increased migration of 60% was observed in *SPRY1 *siRNA transfected cells compared to control cells (Fig 4E).

To further characterize the effect of *SPRY1 *on angiogenesis, we performed a Matrigel tube formation assay on *SPRY1-*siRNA-duplex- and control-siRNA-duplex-transfected cells. When plated on Matrigel, endothelial cells develop into a network of capillary-like vessels and thus provide an *in vitro *model of capillary formation. When tested, both control and *SPRY1-*silenced cells formed networks of tube-like vessels after seeding them on Matrigel in serum containing medium. However, cells transfected with *SPRY1 *silencing siRNA showed an increased network complexity as determined by the number of intersections (Fig 4F). All together these results indicate that the presence of *SPRY1 *expression in endothelial cells prevents angiogenesis.

### *SPRY1 *silencing increases MAPK activation and endothelial cell proliferation by adapting cell-cycle regulator expression

**Figure 5:**
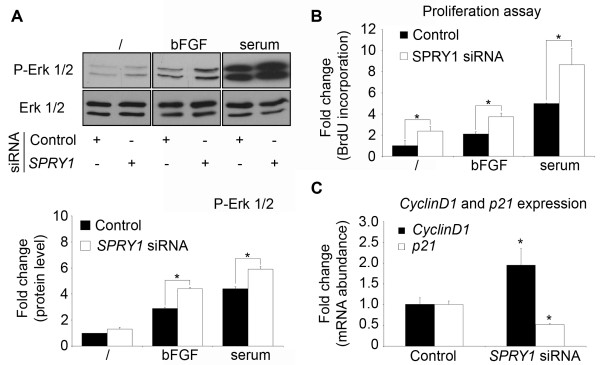
***SPRY1 *silencing increases MAPK activation and endothelial cell proliferation by adapting cell-cycle regulator expression**. ABAE cells were transfected with non-silencing (Control) or *SPRY1 *siRNA for 48 h. **A**. Total protein was extracted from these cells and the level of phospho-Erk1/2 protein was measured by Western blotting. The total level of Erk1/2 protein was used as an internal control. Bottom panel: quantification of the top panel. A densitometry analysis was performed using ImageJ software showing phospho-Erk1/2/total Erk1/2 intensity. **B**. Proliferation was assayed 48 hours after transfection by measuring BrdU incorporation. **C**. *cyclinD1 *and *p21 *mRNA expression quantified by qRT-PCR from ABAE cells 48 h post-tranfection. Data were normalized to the level of *GAPDH *transcript. Mean fold change versus control is shown with the SD (line above the bar, n = 3), *: significance at p < 0.05. The results shown are representative of at least three independent cell transfections.

The last angiogenic process we investigated is one of the most important ones namely endothelial cell proliferation. The inhibitory effect of *SPRY1 *on growth-factor-induced MAPK activation has been widely demonstrated. SPRY1 and SPRY2 are reported to inhibit bFGF-induced tyrosine kinase receptor signal transduction by inhibiting the pathway leading to activation of p42/44 MAPK [27]. We thus examined the effect of *SPRY1 *knockdown on p42/44 MAPK activity in endothelial cells. ABAE cells were transfected with the *SPRY1 *or control siRNA duplex, and were stimulated, after serum starvation, with 10 ng/ml bFGF or 10% serum for 20 minutes. MAPK activation was monitored by immunoblotting with an antibody directed specifically against the phosphorylated forms of p42/44 ERK. As expected, we observed an increased level of phosphorylated p42/44 ERK after bFGF or serum addition. In these conditions, *SPRY1*-knockdown cells showed a significantly higher level of p42/44 ERK phosphorylation than the control cells. The overall level of p42/44 ERK appeared unaffected, as determined by probing with an antibody recognizing all forms of p42/44 ERK (Fig 5A).

Sustained activation of the ERK/MAPK signaling pathway is crucial to allow cell cycle progression and is associated with the induction of positive regulators of cell proliferation and inactivation of cell cycle inhibitors [28]. Having shown that *SPRY1 *decreases ERK/MAPK activation, we examined if *SPRY1 *knockdown really stimulates endothelial cell proliferation. Therefore, transfected ABAE cells were serum starved and then treated with bFGF or serum to induce cell proliferation. The cells responded well to these proliferation stimuli by showing a respectively two-fold and five-fold increase in cell proliferation. Transfection of ABAE cells with *SPRY1 *siRNA duplex increased proliferation of these cells even more as compared to cells transfected with the control siRNA duplex (Fig 5B).

Cell proliferation is controlled by the activity of cyclin-dependent kinases (CDKs), their essential coactivating enzymes, cyclins and CDK inhibitors. Cyclin levels rise and fall during the cell cycle, periodically activating CDKs. Different cyclins are required at different phases of the cell cycle. The three D-type cyclins (cyclins D1, D2, and D3) act as essential sensors which respond to mitogenic stimulation and, upon associating with CDKs, allow cell entry into the G1 phase [29]. Among the different D-type cyclins, activation of the ERK/MAPK pathway is known to allow transcription of the *cyclinD1 *gene [28]. Having shown that *SPRY1 *inhibition increases cell proliferation and MAPK activation, we monitored *cyclinD1 *expression in *SPRY1*-knockdown and control endothelial cells. After serum starvation, transfected ABAE cells were treated with serum for 24 h. Then, RNA was extracted from the transfected cells and subjected to qRT-PCR in order to measure the *cyclinD1 *transcript level. This level was found to be significantly higher in the *SPRY1*-knockdown cells (Fig 5C).

Among the inhibitors of CDKs, the Cip/Kip-family proteins p21, p27, and p57 can interact with a broad range of cyclin-CDK complexes. These inhibitors inactivate CDK-cyclin complexes and are essential to the cell cycle arrest in a broad range of cell types [30]. Moreover, p21 has been demonstrated to be regulated by the MAPK/ERK signaling pathway [31]. This led us to study the effect of *SPRY1 *knockdown on *p21 *expression in ABAE cells. Expression of *p21 *was found to be decreased in *SPRY1*-knockdown than in control cells when cells were cultured in serum containing medium for 24 h after serum starvation (Fig 5C). These results clearly show that SPRY1 negatively regulates endothelial cell proliferation, an important process during new vessel formation.

## Discussion

Since the emergence of angiogenesis as a crucial step in tumor growth and metastasis, great efforts have been made to discover new angiogenesis regulators. In order to identify new genes that control angiogenesis, we previously performed a transcriptomic analysis on endothelial cells after treatment with the potent angiogenesis inhibitor 16 K hPRL [23]. In the list of 16 K hPRL upregulated genes we found *SPRY1*, earlier described as a regulator of branching during trachea development in Drosophila [3]. As angiogenesis is morphologically somewhat similar to branching of the Drosophila tracheal system, *SPRY1 *appeared to be a good candidate. In addition, SPRY1 is a strong inhibitor of growth-factor-induced MAPK signaling required for angiogenesis [27,32] and SPRY1 was demonstrated to block endothelial cell proliferation and differentiation by inhibition of ERK/MAPK signaling induced by bFGF and VEGF [24]. Moreover, SPRY2 and SPRY4, two other SPRY-family members, are reported to play a role in angiogenesis [8,9,33]. Based on these data, we hypothesized that SPRY1 might be an endogenous angiogenesis inhibitor and we therefore decided to study its properties in several angiogenesis models, including tumor-induced angiogenesis in mice.

The results of the present study corroborate our hypothesis. We first confirmed *in vitro *that treatment with the angiostatic agent 16 K hPRL stimulates *SPRY1 *expression both on transcript- and protein-levels. We further demonstrated in our xenograft tumor model that 16 K hPRL specifically enhanced the transcript-level of *SPRY1 *in the (murine) vascular compartment. These data might be very useful in future cancer treatment since *SPRY1 *expression is repressed during tumor development as shown in prostatic and breast cancers [34,35]. Therefore, the re-expression of *SPRY1 *when tumor growth is abolished might be a powerful tool to monitor tumor response to angiostatic treatment or to decide on treatment strategies.

We further show that *SPRY1 *silencing activates endothelial cells to proliferate, adhere to ECM proteins like fibronectin and vitronectin, to migrate, and to form complex vascular networks in a capillary-like-tube formation assay. In addition, *SPRY1 *silencing protects endothelial cells from apoptosis. All these processes are highly relevant to angiogenesis. At least some of the observed effects of *SPRY1 *knockdown might be linked to the previously described role of SPRY1 as an inhibitor of the MAPK pathway [32,36]. Effectively, some reports have already linked MAPK/ERK to cell migration. Pintucci notably highlighted the necessity of ERK1/2 activation for bFGF-induced endothelial cell migration [37]. In line with these data, we observed an increased ERK1/2 activation and a higher migration capacity in *SPRY1*-silenced cells. Moreover, SPRY2, a family member of SPRY1, has been shown to inhibit migration of tumor cells in response to serum and several growth factors [38]. They also demonstrated that the anti-migratory effect of SPRY2 is mediated by the inhibition of Rac1 activation in epithelial cells [39]. According to our data, SPRY1 seems to have similar effects to SPRY2 on endothelial cell migration. However, further studies are still needed to clarify whether Rac1 inhibition is also involved in the anti-migratory action of SPRY1.

The adhesion of endothelial cells to the ECM plays a major role in cell migration. To date, the potential involvement of SPRY1 in endothelial cell adhesion to ECM proteins has never been studied. According to our results, deletion of *SPRY1 *potentiates adhesion of endothelial cells to fibronectin and vitronectin. The differential adhesion to vitronectin might be related to the MAPK/ERK signaling as well. Previous reports have shown in osteoblasts that inhibition of MAPK/ERK signaling decreases adhesion of these cells on different substrates, including vitronectin [40]. This was accompanied by a reduction of α_v_β_3 _integrin expression which was shown to mediate adhesion to vitronectin. Adhesion to fibronectin has also been shown to be dependent on MAPK/ERK activation [41].

Proteins of the Sprouty family, like SPRY2, have been demonstrated to possess anti-apoptotic properties. Edwin and coworkers notably demonstrated that silencing of *SPRY2 *abolishes the anti-apoptotic action of serum in adrenal cortex adenocarcinoma cells [42]. Moreover, SPRY2 has also been implicated in the inhibition of UV radiation-induced apoptosis in HRas-transformed human fibroblasts [43]. Here, we reported a pro-apoptotic effect for SPRY1, suggesting differential roles for SPRY1 and SPRY2 in controlling apoptosis. However, in a few cases, SPRY2 has been attributed to pro- apoptotic capacities such as in differentiated neuronal cells [44]. On the other hand, apoptosis can also be regulated by the MAPK pathway, as demonstrated by Gupta, who showed that VEGF protects HDMECs from apoptosis by activating MAPK/ERK signaling [45]. The pro-apoptotic role of *SPRY1 *deduced from our study may thus be due to *SPRY1*-mediated inhibition of MAPK signaling.

To understand how *SPRY1 *regulates cell proliferation, we examined the MAPK related factors *p21 *and *cyclinD1*, whose products respectively downregulate and upregulate cell cycle progression [29,46]. The regulation of *p21 *by the ERK signaling pathway however, has been under debate. In some cases, ERK signaling induces p21 accumulation, as demonstrated in chondrocyte maturation [47]. Other studies have highlighted the importance of ERK1/2 inhibition in inducing *p21 *expression. For example, Han and coworkers reported that fibronectin induces lung cancer carcinoma cell proliferation by activation of the MAPK pathway, leading to a reduction in *p21 *expression [48]. Moreover, terbinafin-induced cell-cycle arrest through an up-regulation of p21 in HUVECs was shown to be mediated by the inhibition of ERK activation [31]. We demonstrated here that the induction of cell proliferation by *SPRY1 *silencing in endothelial cells is associated with increased *cyclinD1 *and reduced *p21 *transcript levels. Therefore, our results reinforce the inhibitory role of ERK1/2 in the regulation of *p21*.

The results we obtained here are in line with the effects we previously showed for the potent angiostatic agent 16 K hPRL which was used to identify *SPRY1*. Similar to *SPRY1 *which is upregulated by 16 K hPRL, Tabruyn et al. demonstrated that 16 K hPRL induces endothelial cell-cycle arrest in association with a decrease in c*yclinD1 *expression and the induction of *p21 *[21]. In addition we showed that *SPRY1 *expression induced by 16 K hPRL requires NF-κB activation like the angiostatic protein 16 K hPRL. Therefore we attempted to connect the effects of 16 K hPRL on endothelial cells to SPRY1. However, 16 K hPRL still induces apoptosis and inhibits proliferation after *SPRY1 *silencing (data not shown). Thus, SPRY1 does not seem to be essential for the induced apoptosis or decreased proliferation by 16 K hPRL. According to the microarray data previously obtained [23], these results are not surprising. The transcriptomic study revealed 216 transcripts differentially expressed after 2 h of 16 K hPRL treatment. So it could be predicted that suppression of only one target gene of 16 K hPRL would not be able to completely abolish the effects of 16 K hPRL. Nevertheless, the fact that endothelial cells respond opposite to treatment with *SPRY1 *siRNA, regarding proliferation and apoptosis, compared to 16 K hPRL treatment indicates that *SPRY1 *might be involved in the effects of 16 K hPRL.

## Conclusions

In summary, we have shown here that down-regulation of endogenous *SPRY1 *increases angiogenesis-related processes in endothelial cells. *SPRY1 *silencing notably enhances endothelial cell proliferation, a finding possibly linked to SPRY1-mediated modification of *p21 *and *cyclinD1 *expression and/or inhibition of RTK-induced MAPK activation. Involvement of SPRY1 in endothelial cell adhesion to ECM proteins was demonstrated here for the first time. In addition, we show *in vivo *an endothelial cell specific increase of *SPRY1 *expression after treatment with an angiostatic agent. This all strengthens our conclusion that SPRY1 acts as an angiogenesis inhibitor and makes it an interesting target for future cancer therapies. Since, if *SPRY1 *silencing enhances tumor angiogenesis, then restoring *SPRY1 *expression should be an interesting way to reduce tumor growth.

## Methods

### Production of recombinant protein and chemical compounds

Recombinant 16 K hPRL was produced and purified from *E. Coli *as previously described [20]. The purity of the recombinant protein exceeded 95% (as estimated by Coomassie blue staining) and the endotoxin level was found to be 0.5 pg/ng recombinant proteins, as quantified with the "Rapid Endo Test" from the European Endotoxin Testing Service (Lonza, Verviers, Belgium). BAY 1170-82 was purchased from Calbiochem (La Jolla, CA).

### Cell cultures

ABAE (Adult Bovine Aortic Endothelial) cells were isolated as previously described [49]. The cells were grown in low-glucose DMEM containing 10% fetal bovine serum (FBS) and 100 U/ml penicillin/streptomycin. Recombinant bFGF (Promega) was added (1 ng/ml) to the culture every other day. Confluent cells corresponding to passages 8 to 13 were used in the experiment. HMVEC (Human Microvascular Endothelial cell) cultures were maintained in EBM2 medium (Lonza, Walkersville, Walkersville, USA) containing 0.1% hEGF, 0.04% hydrocortisone (kit EGM-2 SingleQuots, Cambrex Bio Science Walkersville, Walkersville, USA), 10% FBS, and 100 U/ml penicillin/streptomycin. HCT116 cells (human colorectal carcinoma cells) were grown in McCoy's 5a medium containing 10% FBS and 100 U/ml penicillin/streptomycin. HEK 293 (Human Embryonic Kidney) cells and adenovirus-E1-transformed HEK 293 cells (BD Biosciences, San Diego, CA) were grown in DMEM supplemented with 10% fetal calf serum (FCS), 1% non-essential amino acids, 100 U/ml penicillin/streptomycin, and 2.5 μg/ml fugisone.

### Adenovirus vectors

16 K-Ad is a defective recombinant E1-E3-deleted adenovirus vector encoding a secreted peptide consisting of the first 139 amino acids of PRL. This adenovirus vector was constructed as described in [16] with the help of the Adeno-X expression system (BD Biosciences, Erembodegem, Belgium). Briefly, the 16 K hPRL complementary DNA was cloned into a pShuttle vector in an expression cassette, which was then inserted into the Adeno-X viral DNA. Recombinant adenoviruses were constructed and amplified in HEK 293 cells. The BD Adeno-X Virus Purification kit (BD Biosciences, Erembodegem, Belgium) and the Adeno-X Rapid Titer Kit (BD Biosciences, Erembodegem, Belgium) were used to perform purification and titration, respectively, of the recombinant adenoviruses (according to the manufacturer's instructions). Null-Ad is a control adenovirus carrying an empty expression cassette.

### Mice

Adult female NMRI nude mice (6-8 weeks of age) purchased from Janvier Breeding (Elevage Janvier, France) were used for tumor growth experiments. The animal experiment protocol used was approved by the Institutional Ethics Committee of the University of Liege.

### Mouse xenograft tumor model

Subconfluent HCT116 cells were trypsinized, washed, and resuspended in PBS. Cell suspension (3.10^6 ^cells in 50 μl PBS) was injected *s.c*. into the right flank of NMRI nude mice 2 weeks before the first adenovirus administration. Sixteen mice were used and randomly divided into two groups of 8 mice. Mice received four intratumoral injections of 5.10^8 ^pfu 16 K-Ad or Null-Ad (control) starting when the HCT116 tumors reached 150 mm^3^. These injections were repeated every 2 days. Ten days after the first adenoviral vector injection, the mice were euthanized and their tumors harvested. Tumor growth was assessed by measuring the length and width of each tumor every 2 or 3 days and calculating tumor volume by means of the formula: length × width^2 ^× 0.5 [50].

### SiRNA Transfections

Small interfering RNA (siRNA) duplexes were obtained from Integrated DNA Technologies (Integrated DNA Technologies, Coralville, USA), two targeting *SPRY1 *and one negative control. Cells were transfected by the CaPO_4 _method. Briefly, 90,000 ABAE cells were seeded into a 6-well plate and allowed to adhere overnight. One hour before transfection, the medium was replaced with fresh medium without antibiotics. SiRNA-CaCl_2 _complexes were made by first combining siRNA with 10 μl of 2.5 M CaCl_2_. One hundred microliters of HSBP (280 mM NaCl, 1.9 mM Na_2_HPO_4_; 12 mM glucose, 10 mM KCl; 50 mM Hepes, pH 7.05) were added and the mix was incubated for one minute at room temperature. Next the mix was added dropwise to the cells followed by an incubation period of 16 h. Cells were then collected and seeded for further tests.

### Quantitative real time PCR (qRT-PCR) analysis

Total RNA was extracted with the RNeasy Mini Kit (QIAGEN) according to the manufacturer's instructions. Synthesis of cDNA was performed from 1 μg total RNA, which was reverse transcribed with the Transcriptor First Strand cDNA Synthesis Kit (Roche, Clinical Laboratories, Indianapolis, IN) according to the manufacturer's instructions. The resulting cDNA was used for quantitative real-time PCR with the one-step 2× Mastermix (Diagenode, Liège, Belgium) containing SYBR green. Thermal cycling was performed on an Applied Biosystem 7000 detection system (Applied Biosystems, Foster City, CA). No-template controls were run for all reactions, and random RNA preparations were also subjected to sham reverse transcription to check for the absence of genomic DNA amplification. The relative transcript level of each gene was obtained by the 2^-ΔΔCt ^method [51] and normalized with respect to the housekeeping gene glyceraldehyde-3-phosphate dehydrogenase (GAPDH) (*in vitro *assays) or cyclophilin A (PPIA) (mouse assays). Primers were designed with the Primer Express software and selected so as to span exon-exon junctions to avoid detection of genomic DNA (see Additional file 3 - List of primers used in quantitative RT-PCR). In order to verify species specificity of the PCR, PCR combining human or mouse cDNAs with human or mouse primers have been performed on cloned cDNAs for PPIA or Sprouty obtained form the German Resource Center for Genome Research (RZPD, IMAGENES, Germany). For analysis by end-point PCR, the final products of the qRT-PCR obtained after 40 cycles of PCR was loaded on agarose gel for electrophoresis.

### Preparation of cell extracts

Cells were washed twice with cold PBS and scraped into lysis buffer [25 mM HEPES (pH 7.9), 150 mM NaCl, 0.5% Triton, 1 mM dithiothreitol, 1 mM phenylmethylsulfonylfluoride] on ice. Insoluble cell debris was removed by centrifugation at 10000 × *g *for 15 min. Aliquots of protein-containing supernatant were stored at -80°C. Protein concentrations were determined by the Bradford method, with the Bio-Rad protein assay reagent (Bio-Rad Laboratories, Inc., Hercules, CA).

### Western blot analysis

Soluble cell lysate (30 μg) was resolved by SDS-PAGE (12%) and transferred to a polyvinylidene fluoride membrane (Milipore Corp., Bedford, MA). Blots were blocked overnight with 8% milk in Tris-buffered saline with 0.1% Tween 20 and probed for 1 h (or 2 h at 37°C for anti-SPRY1 antibody) with primary antibodies: anti-Prolactin A602 (home-made), anti-SPRY1 (Santa Cruz Biotechnology, Santa Cruz, CA, USA), anti-phospho-p44/42 Map Kinase (Thr202/Tyr204) antibody (Cell Signaling Technology, Beverly, MA), anti-MAP Kinase 1/2 (Millipore, Billerica, MA), polyclonal rabbit anti-beta-tubulin (Abcam plc, Cambridge CB4 OFW, UK). After three washes with Tris-buffered saline containing 0.1% Tween 20, antigen-antibody complexes were detected with peroxidase-conjugated secondary antibody and an enhanced fluoro-chemiluminescent system (ECL-plus; Amersham Biosciences, Arlington Heights, IL).

### Immunostaining

ABAE cells were fixed with paraformaldehyde 1% for 30 min and permeabilized with 0.2% Triton X-100 in PBS for 5 min. The samples were blocked with 0.2% bovine serum albumin in PBS for 30 min and incubated with rabbit anti-SPRY1 over night at 4°C. This was followed by incubation with a goat anti-rabbit-Cy3 for 30 min. Fluorescence was analyzed with an Olympus fluorescence microscope and a camera linked to the Analysis software (Soft Imaging System GmbH, Münster, Germany).

### Caspase-3 activity assay

Control and SPRY1-siRNA-transfected cells were plated in 24-well culture plates at a density of 20,000 cells per well in 500 μl of 10% FCS/DMEM. Caspase-3 activity was measured 48 h post-transfection with the CaspACE Assay System Fluorimetric (Promega Corp., Madison, WI) according to the manufacturer's instructions.

### Analysis of cell proliferation

Transfected cells were plated in 96-well culture plates at a density of 5,000 cells per well in 10% FCS/DMEM and allowed to adhere for 6 h. Following this, complete medium was replaced with DMEM free for 24 h. The transfected cells were then incubated in 10% FBS/DMEM or DMEM containing 10 ng/ml bFGF and proliferation was analyzed 24 h later by measuring BrdU incorporation by means of the Cell Proliferation ELISA, BrdU (Colorimetric) (Roche, Clinical Laboratories, Indianapolis, IN)

### Capillary network formation on a Matrigel matrix

The ability of SPRY1-siRNA-transfected ABAE cells to form capillary networks was evaluated in a Matrigel™angiogenesis assay. Briefly, 80,000 cells were plated in 24-well plates coated beforehand with 300 μl Matrigel. Control-siRNA- and SPRY1-siRNA-transfected cells were seeded into 200 μl of DMEM or 10% FBS/DMEM for 16 h. In order to visualize vessels under a fluorescence microscope, the cells were incubated with calcein-AM (2 μM) for 20 min. Quantitative analysis of network structures was performed by measuring the number of connections between vessels in the network. Photographs were taken with an Olympus fluorescence microscope and a camera linked to the Analysis software (Soft Imaging System GmbH, Münster, Germany)

### Migration assay

Eight-micrometer 24-well Boyden chambers (Transwell; Costar Corp, Cambridge, MA) were used for cell migration assays. Both sides of the membrane were coated overnight with 0.005% gelatin. The lower chamber was filled with 600 μl DMEM containing 1% BSA and 10 ng/ml bFGF. ABAE cells transfected with siRNA duplexes, as described above, were placed in 300 μl of 0.1% BSA/DMEM in the upper chamber and allowed to migrate for 16 h at 37°C. After fixation, cells stained with 4% Giemsa were counted on the lower side of the membrane. Cell counting was performed with an ImageJ http://rsbweb.nih.gov/ij/ macro relying on color thresholding in the RGB color space, followed by connected component labeling with the "Analyze Particles" function with size and circularity criteria. The same set of parameters was used for the experiments, and detection masks were produced and double-checked by visual examination.

### Adhesion assay

Cell adhesion experiments were performed in 96-well plates coated with either vitronectin or fibronectin. Wells were coated with 50 μl vitronectin (10 μg/ml) or fibronectin (10 μg/ml) for 1 h, and then washed twice with PBS. Briefly, 50,000 siRNA-transfected cells were plated on the coated 96-well plates and allowed to adhere for 1 h. The wells were then washed twice with medium to remove non-adherent cells. The cells were fixed and stained with 0.01% crystal violet in methanol, then the wells were washed extensively with water and the dye was solubilized in methanol. Quantification was performed by reading the optical density at 550 nm with a microplate reader (Wallac Victor^2^; Perkin Elmer, Norwalk, Finland).

### Luciferase reporter Assays

NF-κB luciferase reporter assays were performed as previously described [20]. Luciferase activity was normalized using the β-galactosidase activity with the β-gal Reporter Gene Assay Kit (Roche).

### Quantification and statistical analysis

Quantification of Western blots was performed using ImageJ software http://rsbweb.nih.gov/ij/. All data are expressed as means ± SD unless stated differently. Analyses for statistical significance (the Mann-Whitney test) were carried out with Prism 4.0 software (GraphPad Software, San Diego, CA, USA). Statistical significance was set at *P *< 0.05.

## Abbreviations

VEGF: vascular endothelial growth factor; RTK: receptor tyrosine kinase; ERK: extracellular signal-regulated kinase; MAPK: mitogen-activated protein kinase; SPRY: Sprouty; 16 K hPRL: 16 kDa N-terminal fragment of human prolactin; bFGF: basic fibroblast growth factor; NF-κB: nuclear factor κB; ABAE: adult bovine aortic endothelial; HMVEC: human microvascular endothelial cell; siRNA: small interfering RNA; GAPDH: glyceraldehyde-3-phosphate dehydrogenase; PPIA: peptidylprolyl isomerase A (cyclophilin A); CDK: cyclin-dependent kinase; ECM: extracellular matrix.

## Competing interests

The authors declare that they have no competing interests.

## Authors' contributions

CS participated in experimental design, performed *in vitro *studies and statistical analysis, interpreted the data and wrote the manuscript. AC carried out the experimental design for the animal study and performed the analysis of tumor growth. LM performed quantitative RT-PCR on tumor extracts, undertook analysis of primer specificity and participated in data interpretation. ST participated in the design of the study and in revision of the manuscript. IS and JM conceived the study, and participated in experimental coordination and in manuscript revision. KC revised the manuscript. All the authors read and approved the final manuscript.

## Supplementary Material

Additional file 1 ***SPRY2 *expression *in vivo *in a mouse xenograft tumor model after 16 K hPRL treatment**. Analysis of *SPRY2 *mRNA expression by qRT-PCR using mouse-specific primers in RNA extracted from tumors. Data were normalized with respect to the mouse *PPIA *transcript level. *: significant at p < 0.05. Click here for file

Additional file 2 ***SPRY2 *and *SPRY4 *expression after *SPRY1 *silencing**. ABAE cells were transfected with non-silencing siRNA (Control) or with two different *SPRY1 *siRNAs. *SPRY2 *and *SPRY4 *mRNA levels were measured by qRT-PCR 48 hours after transfection. Data were normalized to the *GAPDH *transcript level. Mean fold change versus untreated cells is shown with the SD (line above the bar, n = 3). *: significant at p < 0.05. The results shown are representative of at least three distinct cell transfections. Click here for file

Additional file 3 **List of primers used in quantitative RT-PCR**. Sequences of all primers used in qRT-PCR experiments are listed. Click here for file
