# Supplementary figures and images for: Sprouty1, a new target of the angiostatic agent 16K prolactin, negatively regulates angiogenesis
Source: Mol Cancer. 2010 Sep 2;9:231. doi: 10.1186/1476-4598-9-231 (PMC2944818; doi:10.1186/1476-4598-9-231)

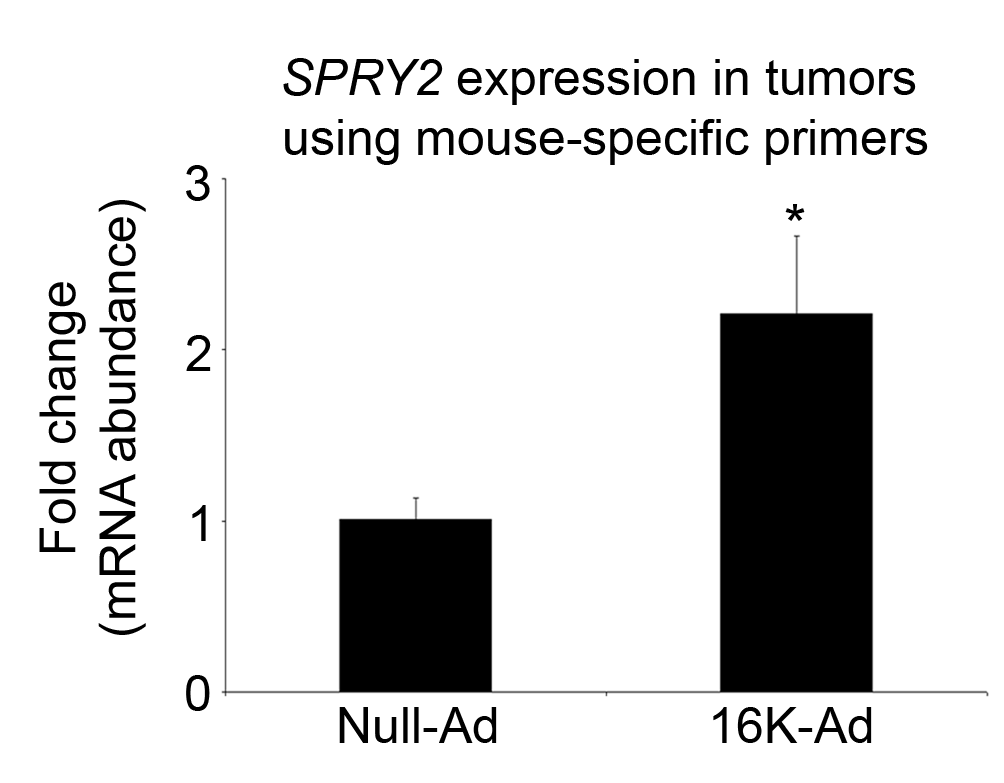

Supplement: Additional file 1 — SPRY2 expression in vivo in a mouse xenograft tumor model after 16 K hPRL treatment. Analysis of SPRY2 mRNA expression by qRT-PCR using mouse-specific primers in RNA extracted from tumors. Data were normalized with respect to the mouse PPIA transcript level. *: significant at p < 0.05. [file 1476-4598-9-231-S1.TIFF]

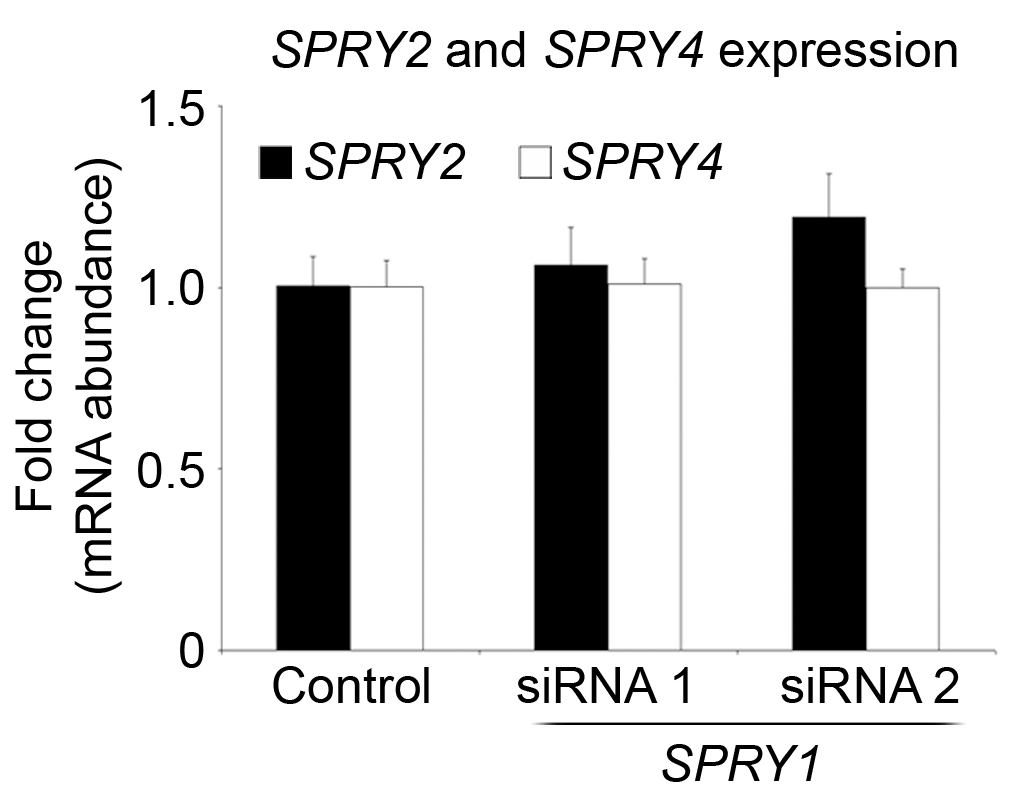

Supplement: Additional file 2 — SPRY2 and SPRY4 expression after SPRY1 silencing. ABAE cells were transfected with non-silencing siRNA (Control) or with two different SPRY1 siRNAs. SPRY2 and SPRY4 mRNA levels were measured by qRT-PCR 48 hours after transfection. Data were normalized to the GAPDH transcript level. Mean fold change versus untreated cells is shown with the SD (line above the bar, n = 3). *: significant at p < 0.05. The results shown are representative of at least three distinct cell transfections. [file 1476-4598-9-231-S2.TIFF]
