# Supplementary material for: Sprouty1, a new target of the angiostatic agent 16K prolactin, negatively regulates angiogenesis
Source: Mol Cancer. 2010 Sep 2;9:231. doi: 10.1186/1476-4598-9-231 (PMC2944818; doi:10.1186/1476-4598-9-231)
Supplement: Additional file 3 — List of primers used in quantitative RT-PCR. Sequences of all primers used in qRT-PCR experiments are listed. [file 1476-4598-9-231-S3.DOC]

**Additional files 1 – List of primers used in quantitative RT-PCR.**

SPRY1 bovine primers

Sense 5’GCATCCTTGGTGGCATACG 3’

Antisense 5’ACCTGGCATGCATCTGAAATC 3’

SPRY1 mouse primers

Sense 5’ GCGGAGGCCGAGGATTT 3’

Antisens 5’ ATCACCACTAGCGAAGTGTGGC 3’

SPRY1 human primers

Sense 5’ GCCTTCTTTGGATAGCCGTCAG 3’

Antisense 5’ TCATTGCTGCCTCTTATGGCC 3’

GAPDH bovine primers

Sense 5’ TGCCCCCATGTTTGTGATG 3’

Antisense 5’ GGTGGTGCAGGAGGCATT 3’

PPIA human primers

Sense 5’ CCAACACAAATGGTTCCCAGT 3’

Antisense 5’ CCATGGCCTCCACAATATTCA 3’

PPIA mouse primers

Sense 5’ ACCGTGTTCTTCGACATCACG 3’

Antisense 5’ CTGGCACATGAATCCTGGAATA 3’

SPRY2 bovine primers

Sense 5’ AGACTGGATCTGCGACAAGCA 3’

Antisense 5’ CGTCGTCGTTGGAACAGTGATA 3’

SPRY3 bovine primers

Sense 5’ GCAAACCCACAAATCTGATTGG 3’

Antisense 5’ TGGCTCAAATGCTGAGGCA 3’

SPRY4 bovine primers

Sense 5’ AGACCGGACAAGCCTTTCTGA 3’

Antisense 5’ GGATGCTAAACCTCTGGCTGTG 3’

CyclinD1 bovine primers

Sense 5’GCCGAGAAGCTGTGCATTT 3’

Antisense 5’GGAAGTGCTCGATGAAGTC 3’

p21 bovine primers

Sense 5’GGCAGACCAGCATGACAGATT 3’ GGCAGACCAGCATGACAGATT GGCAGACCAGCATGACAGATT GGCAGACCAGCATGACAGATT

Antisense 5’AAAAGGTACAGAAGAGGCCCG 3’ AAAAGGTACAGAAGAGGCCCG

SPRY2 mouse primers

Sense 5’ TGCACATCGCTGGAAGAAGA 3’

Antisense 5’ AGGTCTTGGCAGTGTGTTCA 3’
